# MicroRNA-208a Increases Myocardial Fibrosis via Endoglin in Volume Overloading Heart

**DOI:** 10.1371/journal.pone.0084188

**Published:** 2014-01-02

**Authors:** Bao-Wei Wang, Gong-Jhe Wu, Wen-Ping Cheng, Kou-Gi Shyu

**Affiliations:** 1 School of Medicine, Fu-Jen Catholic University, New Taipei City, Taiwan; 2 Division of Cardiology, Shin Kong Wu Ho-Su Memorial Hospital, Taipei, Taiwa; 3 School of Medicine, College of Medicine, Taipei Medical University, Taipei, Taiwan; 4 Department of Anesthesiology, Shin Kong Wu Ho-Su Memorial Hospital, Taipei, Taiwan; 5 Graduate Institute of Clinical Medicine, Taipei Medical University, Taipei, Taiwan; University of Central Florida, United States of America

## Abstract

MicroRNA-208a (mir-208a) is essential for cardiac hypertrophy and fibrosis. Endoglin, a co-receptor of transforming growth factor-β is also essential for cardiac fibrosis. Endoglin has been shown to be a target of mir-208a in the in vitro mechanical stress model. Volume overload can lead to heart failure and cardiac fibrosis. The role of mir-208a and endoglin in volume overload heart failure is well known. We sought to investigate the mechanism of regulation of mir-208a and endoglin in volume overload-induced heart failure. Aorta-caval (AV) shunt was performed in adult Sprague-Dawley rats to induce volume overload. Heart weight and heart weight/body weight ratio significantly increased in AV shunt animals. AV shunt significantly increased left ventricular end-diastolic dimension as compared to sham group. Mir-208a was significantly induced by AV shunt from 3 to 14 days. Endoglin, myosin heavy chain-β and brain natriuretic peptide were significantly induced by AV shunt from 3 to 14 days. Overexpression of mir-208a in the sham group without AV shunt significantly increased endoglin expression similar to the AV shunt group. Antagomir-208a attenuated the endoglin expression induced by AV shunt. Pretreatment with atorvastatin also attenuated the endoglin expression induced by AV shunt. AV shunt significantly increased myocardial fibrosis as compared to sham group. Overexpression of mir-208a in the sham group significantly increased myocardial fibrosis. Antagomir-208a and atorvastatin significantly attenuated the myocardial fibrosis induced by AV shunt. In conclusion, mir-208a increased endoglin expression to induce myocardial fibrosis in volume overloaded heart failure. Treatment with atorvastatin can attenuate the myocardial fibrosis induced by volume overload through inhibition of endoglin expression.

## Introduction

Cardiac fibrosis is closely associated with heart failure because cardiac fibrosis may cause the loss of normal cardiac function [1]. Endoglin is a homeodimeric membrane glycoprotein that is a co-receptor of transforming growth factor-β1 (TGF-β1) and β3 [2]. Endoglin is a potent mediator of profibrotic effects of angiotensin II on cardiac fibroblasts [3] and can modulate the effect of TGF-β1 on extracellular matrix synthesis [4]. These data indicate that endoglin plays an important role in fibrogenesis in cardiac remodeling.

A microRNA (mir) is small, 22-nucleotide non-protein-coding RNA that inhibits transcription or translation by interacting with the 3′untranslated regions of target mRNA and promoting target mRNA degradation (gene silencing) [5]. Recently, Place et al. have demonstrated mir functioning to induce gene expression [6]. Because of their capability to monitor the expression levels of the genes that control both adaptive and maladaptive cardiac remodeling processes, mirs may be vitally involved in the pathogenesis of heart failure [7], [8]. Mir-208a seems to be fundamental for the expression of genes involved in cardiac fibrosis and hypertrophic growth [9], [10]. Mir-208a is upregulated in pressure overloading with thoracic aortic banding [9] and is activated by mechanical stress [11]. The role of mir-208a in volume overload is not known. Mir-208a can increase endoglin expression in cardiac myoblast [11]. Endoglin expression is increased in patients with heart failure [12]. Since volume overload can lead to heart failure and myocardial fibrosis, we sought to investigate the regulation of mir-208a in volume overloading heart.

## Materials and Methods

### The aorta-caval shunt (AV shunt) rat model

AV shunt was performed on rats to induce volume overload. On the day of surgery, the Sprague-Dawley rats weighing 280 to 330 g were anesthetized with 2% isoflurane and the vena cava and aorta were exposed via abdominal midline incision after confirming a fully anaesthetized state (.e.g. no response to toe pinching). The aorta-caval shunt was produced as previously described [13]. Sham-operated control animals were prepared similar manner, except that the aorta was not punctured. Atorvastatin at 30 mg/kg was given by oral gavage for 2 weeks after induction of AV shunt. After 2 weeks of AV shunt induction, rats were euthanized with an overdose of isoflurane. Left ventricular tissue was obtained for Western blot analysis and immunohistochemical staining. Masson's trichrome staining was performed to delineate fibrosis tissue from viable myocardium. All study protocols were approved by our Committee of Animal Care and Use of Shin Kong Wu Ho-Su Memorial Hospital (permit number:091217021) and were carried out in accordance with the Guide for the Care and Use of Laboratory Animals (NIH publication No. 86-23, revised 2011).

### Hemodynamic monitor

Hemodynamic monitor of rats was performed with polyethylene catheters to measure through a Grass model tachogragh preamplifier as previously described [13].

### Assessment of cardiac hypertrophy and function

Cardiac function of rats was evaluated noninvasively by echocardiography performed with an Acuson Sequoia 512 machine using a 15-MHz probe at the day of sacrifice, 7 and 14 days (AV shunt) after the surgery as previously described [13]. The sonographer was blinded to the randomization of rats.

### Construction and delivery of mir-208a expression vector

A 71 bp rat-mir-208a precursor construct was generated as follows. Genomic DNA was amplified with forward primer, CAACAGAAGTGCTTGGAAG and reverse primer, GGCTGATCGACGGTAGCT. The 165 bp amplified product was digested with EcoRI and BamHI restriction enzymes and ligated into pmR-ZsGreen1 plasmid vector (coexpression mir-208 and green fluorescent protein, Clontech Laboratories, Mountain View, CA, USA) digested with the same enzymes. The constructed plasmid (co-expression mir-208 and green fluorescent protein) was transfected into left ventricular myocardium using a low pressure-accelerated gene gun (Bioware Technologies, Taipei, Taiwan) essentially following the protocol from the manufacturer. In brief, 2 mg of plasmid DNA was suspended in 5 ml of PBS and then 100 µl was added to the loading hole near the nozzle. Pushing the trigger of the low pressure gene gun released the DNA-containing solution, which was directly propelled by helium at a pressure of 15 psi into left ventricular myocardium of the rat. The distribution of fluorescent image in treated rat was visualized by a dissecting fluorescence microscope with high resolution CCD (HAMAMATSU PHOTONICS, Japan). After 3 days, the rat chest was re-open and the fluorescent image on left ventricular myocardium was detected. If the fluorescent image was able to visualize, it was regarded as a successful transfection. The efficiency of using this method is around 30%.

### Western blot analysis

Western blot was performed as previously described ‘[14]. Monoclonal rat anti-mouse endoglin antibody, polyclonal myosin heavy chain and polyclonal brain natriuretic peptide antibodies (Santa Cruz Biotechnology, Inc., CA, USA) were used. Equal protein loading of the samples was verified by staining monoclonal antibody α-tubulin (Sigma, St. Louis, MI, USA). Signals were visualized by chemiluminenescent detection. All Western blots were quantified using densitometry.

### Quantitative analysis of microRNAs

TaqMan® MicroRNA real-time quantitative assays were used to quantitate mir as previously described [11]. All fold changes between samples were determined using the ΔΔCT method [15]. In brief, each 15 µl RT reaction contained purified 10 ng of total RNA, 3 µl miR-208 RT primer (Applied Biosystems®, Life Technologies, Grand Island, NY, USA), 1×RT buffer (Applied Biosystems), 0.25 mM each of dNTPs, 3.33 U/µl MultiScribe™ reverse transcriptase (Applied Biosystems) and 0.25 U/µl RNase inhibitor (Applied Biosystems). The reactions were incubated in an Applied Biosystems 9700 Thermocycler in a 96-well plate for 30 min at 16°C, 30 min at 42°C, followed by 5 min at 85°C, and then held at 4°C. Each real-time PCR for each microRNA assay (20 µl volume) was carried out in triplicate, and each 20 µl reaction mixture included 1.33 µl of RT product, 10 µl of 2×TaqMan® Universal PCR Master Mix, 1 µM 20×TaqMan MicroRNA assay. The reaction was incubated in an Applied Biosystems 7300 Real-Time PCR System in 96-well plate at 95°C for 10 min, followed by 40 cycles of 95°C for 15 sec and 60°C for 1 min. The expression levels of target mirs were normalized to U6.

### Immunohistochemical analysis

The left ventricle was harvested and fixed in 10% formaldehyde and sliced into 5 µm paraffin sections. For immunohistochemical stain, the slides were postfixed in 4% paraformaldehyde for 20 min, treated in 3% hydrogen peroxide/PBS for 25 min, blocked in 5% normal rabbit serum for 20 min, blocked with biotin/avidin for 15 min each, and incubated with fluorescent isothiocyanate (FITC)-conjugated rat monoclonal anti-endoglin antibody, polyclonal myosin heavy chain antibody (Santa Cruz Biotechnology). For 2 hours at room temperature, biotinylated rabbit-anti mouse IgG at 1∶400 for 30 min, and Vector Elite ABC biotin-avidin-peroxidase complex for 30 min. Sections were then developed with diaminobenzidine and diaminobenzidine enhancer (Vector), counterstained with hematoxylin. Images were examined with a fluorescent microscope.

### In situ hybridization assay

Five-micrometer-thick tissue sections of left ventricular myocardium were mounted on positively charged barrier frame slides, dewaxed in xylenes, and rehydrated through an ethanol dilution series (100% to 25%). Tissue sections were digested with 5 µg/mL of proteinase K for 20 minutes at 37°C to facilitate probe penetration and exposure of miRNA species. To minimize nonspecific binding based on charge interactions, tissues were subjected to a brief acetylation reaction [66 mmol/L HCl, 0.66% acetic anhydride (v/v) and 1.5% triethanolamine (v/v) in RNase-free water]. Then, tissue sections were prehybridized at the hybridization temperature for 30 minutes in prehybridization solution which consisted of 50% deionized formamide, 5× sodium chloride/sodium citrate buffer, 1× Denhardt's solution, 500 µg/mL of yeast tRNA, and 0.01% Tween. The prehybridization solution was replaced with 200 µl of hybridization solution containing 10 pmol of the FAM-labeled LNA miR-208a probe (product sequence 5′-3′, CTTTTTGCTCGTCTTAT, Exiqon, Vedbaek, Denmark) and tissues were incubated for 90 minutes at the hybridization temperature and washed twice for 10 minutes in sodium chloride/sodium citrate buffer.

### Statistical analysis

The data were expressed as mean+SD. Statistical significance was performed with analysis of variance (GraphPad Software Inc., San Diego, CA, USA). The Dunnett's test was used to compare multiple groups to a single control group. Tukey-Kramer comparison test was used for pairwise comparisons between multiple groups after the ANOVA. A value of P<0.05 was considered to denote statistical significance.

## Results

### AV shunt increases heart size

The heart weight and heart weight/body weight ratio significantly increased after AV shunt for 7 and 14 days (Table 1). The heart rate and mean arterial blood pressure did not change significantly. LV end-diastolic and end-systolic dimension significantly increased after AV shunt for 14 days and inter-ventricular septum thickness and left ventricular posterior wall thickness did not significantly change, indicating the volume-overload induced by AV shunt.

**Table 1:** Hemodynamic and echocardiographic parameters.

	Sham	Shunt 7D	Shunt 14D	Sham/miR-208a	Sham/Mut-208a	Shunt7D/Antagomir-208a	Shunt 7D/Mut-208a	Shunt7D/Atorvastatin
N	6	6	6	6	6	6	6	6
Body weight, g	309±16	305±20	314±26	279±11	287±1 9	304±12	307±16	297±21
Heart weight, mg	801±42	895±57*	1104±61**^+^**	821±40	811±22	844±41	882±29	837±38
Heart weight/body weight, mg/g	2.5±0.4	2.8±0.4*	3.49±0.5*	2.9±0.3	2.7±0.6	2.8±0.6	2.9±0.5	2.7±0.5
Heart rate, min	315±27	346±11	304±22	332±31	319±29	332±22	338±21	324±32
MAP, mmHg	82±9	75±8	71±4	80±7	86±9	77±6	84±9	81±11
IVSTd, mm	1.3±0.2	1.2±0.4	1.1±0.6	1.1±0.5	1.2±0.4	1.2±0.2	1.2±0.4	1.2±0.2
LVPWT, mm	1.2±0.2	1.2±0.4	1.0±0.4	1.3±0.2	1.3±0.3	1.3±0.4	1.1±0.4	1.3±0.3
LVEDD, mm	6.5±0.3	6.9±0.4	7.1±0.5*	6.2±0.4	6.4±0.5	6.2±0.5	6.4±0.4	6.3±0.4
LVESD, mm	3.2±0.3	3.6±0.3	4.1±0.4*	3.5±0.3	3.3±0.6	3.2±0.6	3.4±0.5	3.2±0.5
FS, %	47±7	44±6	42±7	45±5	47±5	47±9	45±8	46±8

MAP  = mean arterial pressure. IVSTd =  inter-ventricular septum end-diastolic thickness. LVPWT  =  left ventricular posterior wall thickness. LVEDD  =  left ventricular end-diastolic dimension. LVESD  =  left ventricular end-systolic dimension. FS  =  fraction shortening. *P<0.01 vs. sham.

### AV shunt increases myocardial mir-208a expression

**Figure 1:**
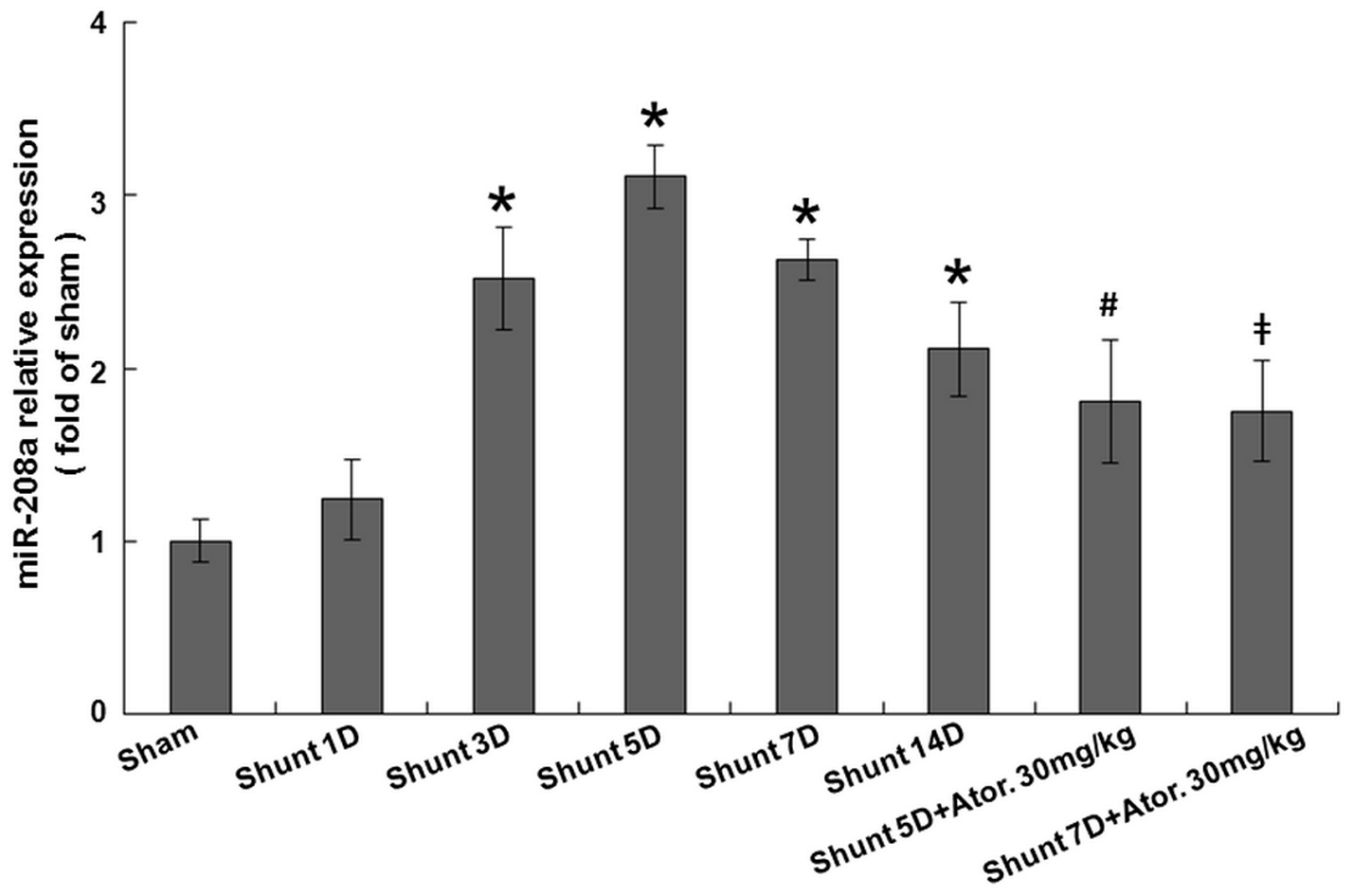
Aorta-caval shunt increases mir-208a expression in rat myocardium. Pretreatment with atorvastatin significantly attenuated the increase of mir-208a expression induced by AV shunt. *P<0.001 vs. sham group (n = 5–6 per group). ^#^P<0.01 vs. shunt 5D. ^‡^P<0.001 vs. shunt 7D.

As shown in Figure 1, AV shunt significantly increased myocardial mir-208a expression at 3 days after shunting, reached a maximal of 3.1±0.2-fold at 5 days and remained elevated for up to 14 days after shunting. Pretreatment with atorvastatin significantly attenuated the increase of mir-208a induced by AV shunt. However, the mir-208a level was still higher in the atorvastatin-treated group than in the sham group, indicating that atorvastatin partially but not completely inhibited the increase of mir-208a expression induced by AV shunt.

### AV shunt increases myocardial endoglin expression

**Figure 2:**
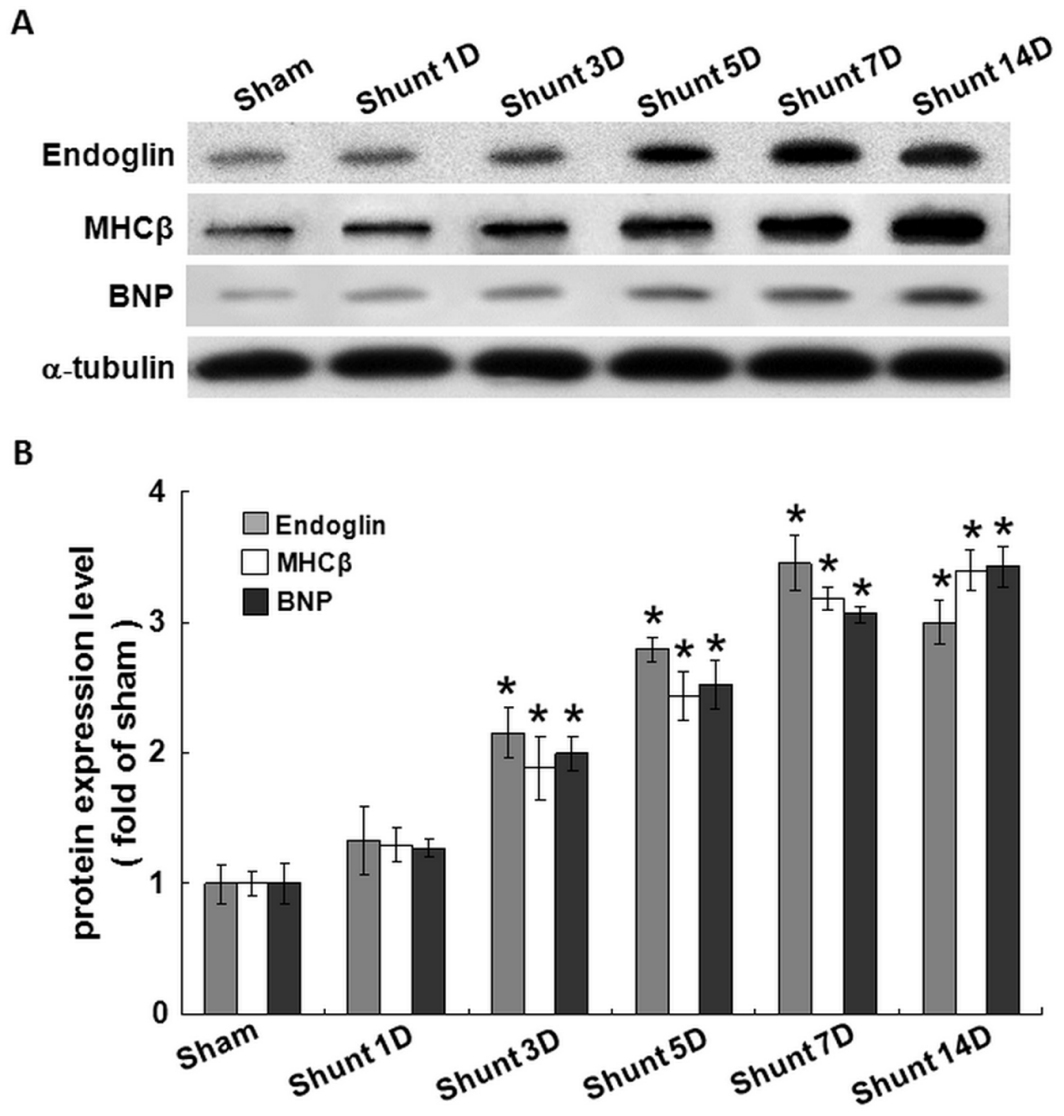
Aorta-caval shunt increases endoglin, β-myosin heavy chain (MHCβ) and brain natriuretic peptide (BNP) proteins expression in rat myocardium. A. Representative western blot for endoglin, MHCβ, and BNP protein expression in rat myocardium after different days of shunting. B, Quantitative analysis of endoglin, MHCβ, and BNP protein levels. The values from myocardium after AV shunt have been normalized to matched α-tubulin measurement and then expressed as a ratio of normalized values to protein in sham group (n = 6 per group). *P<0.001 vs. sham.

As shown in Figure 2, AV shunt significantly increased myocardial endoglin protein expression from 3 days up to 14 days. The cardiac hypertrophic markers such as βMHC and BNP were also significantly induced by AV shunt from 3 to 14 days as the endoglin protein.

### Mir-208a mediates the myocardial endoglin expression

**Figure 3:**
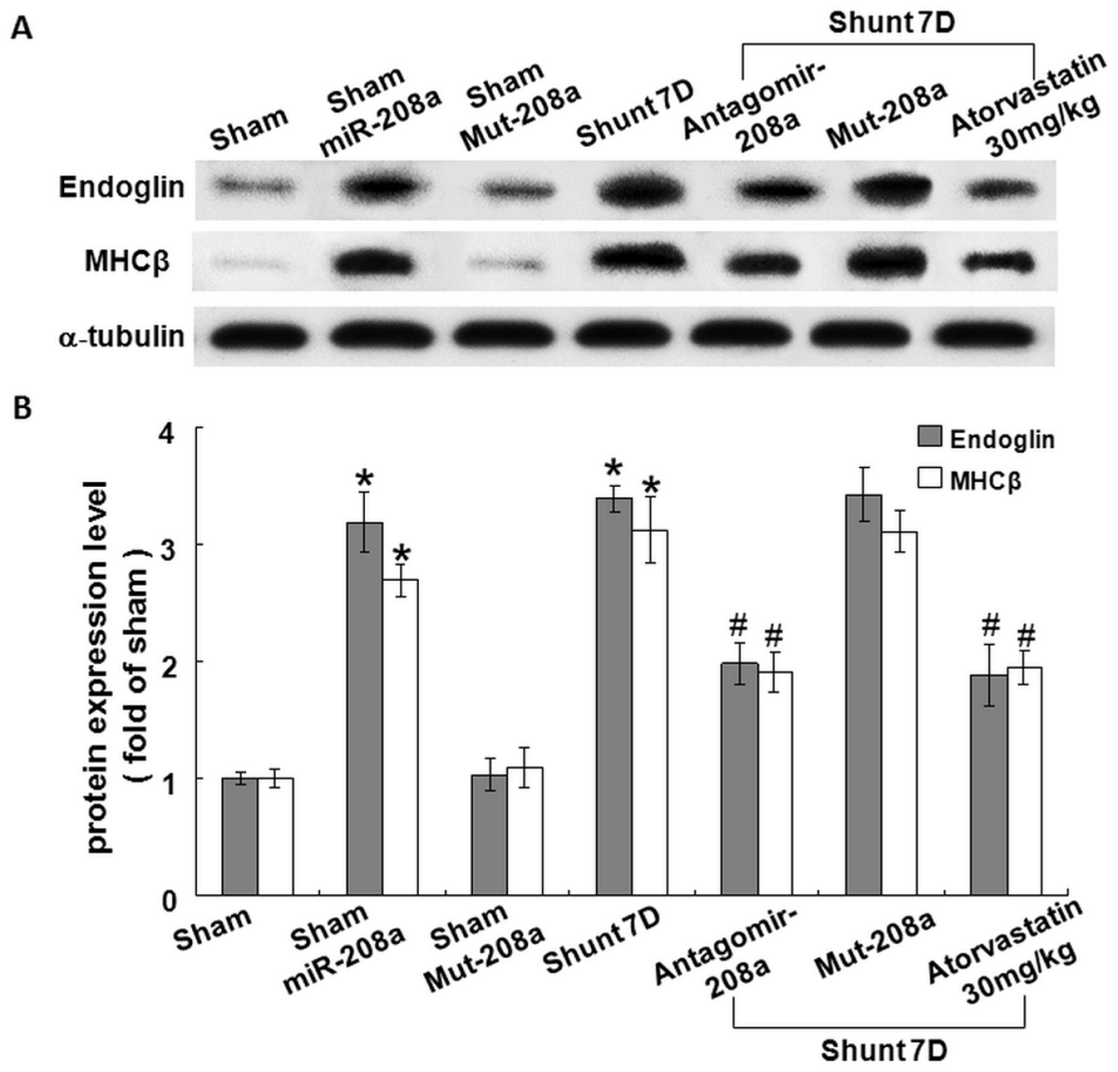
Mir-208a mediates the myocardial endoglin expression in AV shunt rat. A, Representative western blot for endoglin and MHCβ protein expression in the rat myocardium after 7 days of shunting. Mir-208 expression vector was transfected into left ventricular myocardium by low pressure-accelerated gene gun. Overexpression of mir-208a in the sham group significantly increased endoglin and MHCβ protein expression.*P<0.001 vs. sham. #P<0.001 vs. shunt 7D. (n = 6 per group). Pretreatment with atorvastatin significantly attenuated the increase of endoglin and MHCβ protein expression induced by AV shunt.

**Figure 4:**
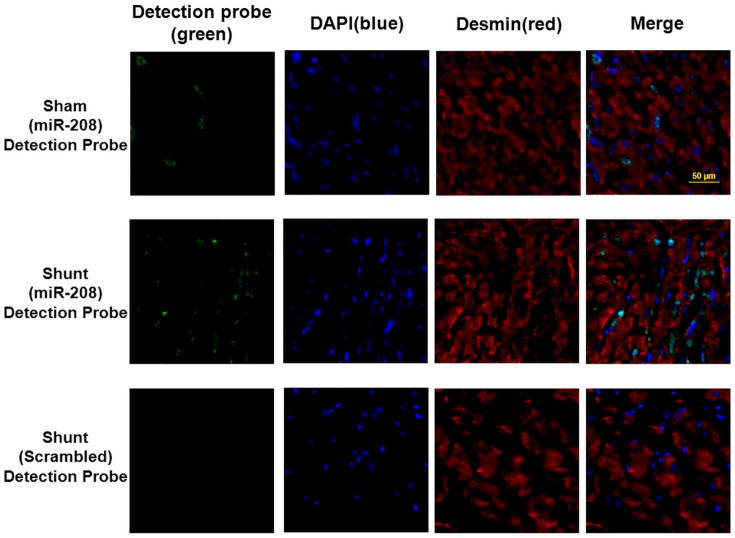
In situ hybridization assay detects the presence of mir-208a in the cardiac myocytes. Representative microscopic images showing the presence of mir-208a (green color) in the cytoplasm of cardiac myocytes from left ventricular myocardium in AV shunt rats. The sham group or scrambled probe did not detect the presence of mir-208a.

**Figure 5:**
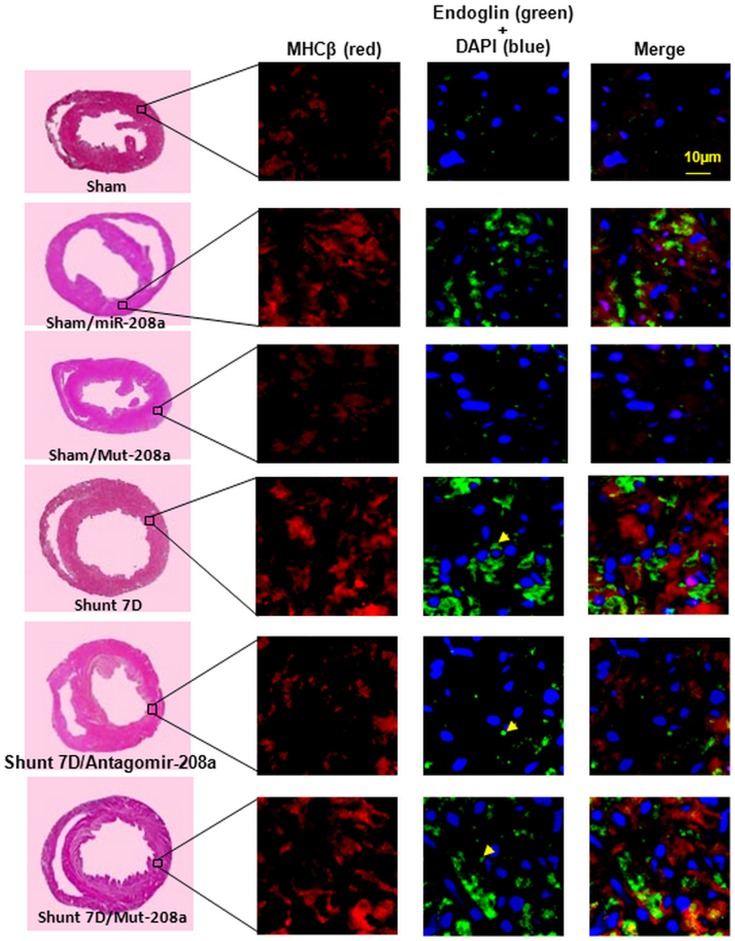
Immunohistochemnical staining of left ventricular myocardium after induction of aorta-caval shunt with or without antagomir-208a treatment. There are significantly increased immunoreactive signals for endoglin and MHCβ after overexpression of mir-208a and AV shunt for 7 days. Antagomir-208a significantly decreased the immunoreactive signal induced by AV shunt. Rare endoglin signals were seen in the sham group.

To investigate the effect of mir-208a on myocardial endoglin expression, over-expression of antogomir208a and mutant type mir-208a (mut-208a) in the left ventricle was performed. AV shunt at 7 days significantly increased myocardial endoglin and βMHC protein expression and over-expression of antagomir208a significantly inhibited the increase of myocardial endoglin and βMHC protein expression induced by AV shunt. Over-expression of mutant mir-208a (mut-208a) did not have the effect on myocardial endoglin and βMHC expression induced by AV shunt. Over-expression of mir-208a in the sham group without AV shunt significantly increased myocardial endoglin and βMHC protein expression while over-expression of mut-208a in the sham group did not induce myocardial endoglin and βMHC protein expression (Figure 3). Pretreatment of atorvastatin significantly attenuated the increase of myocardial endoglin and βMHC protein expression induced by AV shunt (Figure S1). The transfection of mir-208a into myocardium was monitored by a dissecting fluorescence microscope as shown in Figure S2. The presence of mir-208a in the cytoplasm of cardiac myocyte was confirmed by in situ hybridization assay (Figure 4). Immunohistochemical staining showed that increased myocardial endoglin and βMHC expression after AV shunt and over-expression of mir-208a in the sham group (Figure 5). Mutant mir-208a did not change myocardial endoglin and βMHC expression after AV shunt. Myocardial endoglin and βMHC were not stained in the control sham group.

### Mir-208a increases myocardial fibrosis

**Figure 6:**
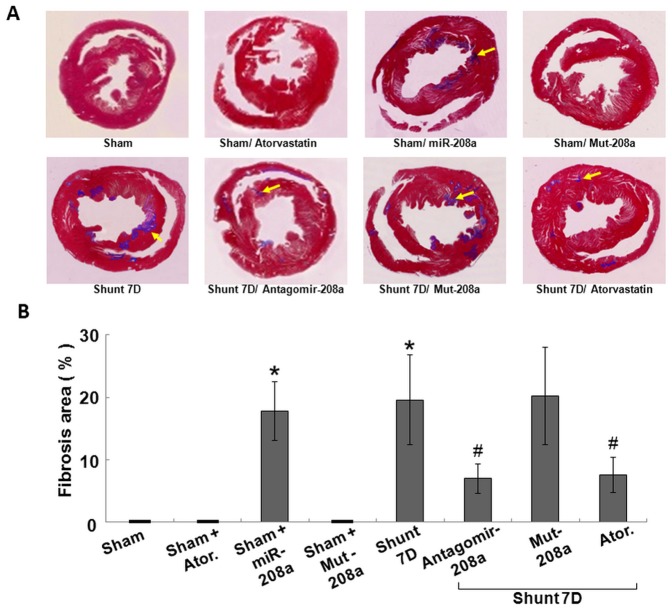
Antagomir-208a and atorvastatin decreases myocardial fibrosis induced by aorta-caval shunt. A. Representative Masson's trichrome stain for cross-section of myocardium. Masson's trichrome staining was performed to delineate fibrosis tissue from viable myocardium. B. Quantitative analysis of fibrosis area. N = 6 per group. *P<0.001 vs. sham group. ^#^P<0.001 vs. shunt 7D.

AV shunt and over-expression of mir-208a in the sham group significantly increased myocardial fibrosis area as compared to sham group (Figure 6). Over-expression of mut-208a in the sham group did not change the fibrosis area as compared to the sham group. Overexpression of antagomir208a and pretreatment with atorvastatin in the AV shunt group significantly decreased myocardial fibrosis area induced by AV shunt. Over-expression of mut-208a in the AV shunt did not decrease the fibrosis area induced by AV shunt. This finding indicates that mir-208a plays a crucial role in the myocardial fibrosis after AV shunt.

## Discussion

Endoglin expression is increased in human hearts with severe left ventricular failure and in heart failure induced by transaortic constriction, a pressure overload in mice [12]. Kapur et al. have demonstrated that reduced endoglin activity by soluble endoglin can limit cardiac fibrosis and improve survival in heart failure [12]. Endoglin also regulates angiotensin-mediated fibrosis via angiotensin receptor [16]. In the present study, endoglin expression is elevated in acute volume-overload heart induced by AV shunt, indicating that endoglin plays a crucial role in pressure- and volume-overload heart failure. Endoglin has been used as a noninvasive measure of left ventricular filling pressure in heart failure [17] and has been regarded as a new biomarker for acute heart failure [18]. Therefore, targeting endoglin to prevent fibrosis and heart failure may improve clinical outcome in patients with heart failure in addition to the current clinical benefits of β-adrenergic receptor antagonists, angiotensin-converting enzyme or receptor blockers and aldosterone antagonists [19]. AV shunt model in our study increased heart weight, heart weight/body weight ratio, and increased left ventricular size without increased septal and posterior wall thickness, indicating an eccentric hypertrophy which is consistent with volume overload heart failure status.

Mirs are reported to be aberrantly expressed in hypertrophic heart [20]. Mir-208 is expressed specifically in the heart with trace expression in the lung [9]. Mir-208a is also essential for the expression of the genes involved in cardiac hypertrophic growth. Although mir-208a was upregulated and cardiac hypertrophy was induced in thoracic aortic banding, a pressure overload model [8], it is not known whether mir-208a is also induced in volume overload. In the present study, we demonstrated for the first time that mir-208a was induced in acute volume overload by AV shunt in rat. Over-expression of mir-208a in the sham group without AV shunt significantly increased myocardial endoglin expression. Over-expression of antagomir208a in the AV shunt group significantly decreased myocardial fibrosis area induced by AV shunt, indicating that mir-208a plays a crucial role in the myocardial fibrosis after AV shunt. The increased endoglin to induce myocardial fibrosis induced by AV shunt was mediated by mir-208a. Bedsides endoglin, βMHC is also a target of mir-208a. Overexpression of mir-208a in cardiac myocytes increases βMHC protein expression and addition of antagomir-208a significantly attenuates the increase of βMHC induced by overexpression of mir-208a [21]. Overexpression of mir-208a did not increase protein expression of thyroid hormone receptor associated protein 1, brain natriuretic peptide and αMHC [21].

Patients with congenital heart disease may have myocardial fibrosis similar to patients with acquired heart failure [22]. In animal model of pulmonary artery banding and trans-valvular patch to induce right ventricular failure and mimic repaired tetralogy of Fallot, myocardial fibrosis was observed in infant piglets [23]. These data indicate that cardiac fibrosis may begin at the embryonic and early infant stage with heart defect causing volume overload. Mir208a may also play a pivotal role in the formation of cardiac fibrosis in congenital heart disease, not just in acquired heart disease. Therapeutic innovation target miR208a to improve cardiac fibrosis may warrant further research.

Statin, a 3-hydroxy 3-methyl glutaryl-CoA reductase (HMG-CoA reductase) inhibitor, improves survival in patients with ischemic and non-ischemic heart failure [24]. Recently, high dose atorvastatin significantly reduces hospitalization for heart failure in patients with stable coronary heart disease [25]. Atorvastatin can reduce endoglin expression in endothelium in apo-E deficient mice and C57BL/6J mice [26], [27]. Statin provides antifibrotic effect via blocking the angiotensin II-mediated oxidative stress and procollagen-1 expression in cardiac fibroblast [28]. Recently, we have demonstrated that atorvastatin can inhibit endoglin expression induced by TGF-β1 in cultured cardiac fibroblast [12]. The antifibrotic effect of statin has also been demonstrated in cardiac myocytes through RhoA-extracellular signal kinase-serum response factor signaling pathway [29]. In this study, we further confirm that atorvastatin can reduce myocardial fibrosis through reducing endoglin expression in volume overloading heart. We have previously demonstrated that TGF-β1 can activate mir-208a expression in cardiac myocytes [21] and atorvastatin can inhibit the TGF-β1 expression. Therefore, the reason that pretreatment of atorvastatin in volume overload model can reduce mir-208a possibly is through the anti-inflammatory or pleiotropic effect of atorvastatin, partially by the anti-TGF-β1 effect. The dose of atorvastatin used in the animal study ranged from 10 mg/kg/day to 50 mg/kg/day [16], [30], [31]. In the present study, we chose 30 mg/kg/day of atorvastatin as the therapeutic dose because several previous studies used this dose [30], [32], [33]. Statin therapy may become another therapeutic strategy for controlling endoglin-associated pathologic cardiovascular disease in humans.

In conclusion, we demonstrate for the first time that mir-208a increases endoglin expression to induce myocardial fibrosis in volume overloaded heart failure. Treatment with atorvastatin can attenuate myocardial fibrosis induced by volume overload through inhibition of endoglin expression.

## Supporting Information

Figure S1 **Immunohistochemnical staining of left ventricular myocardium after induction of aorta-caval shunt with or without atorvastatin treatment.** There are significantly increased immunoreactive signals for endoglin and MHCβ after AV shunt for 7 days. Pretreatment with atorvastatin significantly decreased the immunoreactive signal induced by AV shunt. Rare endoglin signals were seen in the sham group.
(TIF)

Figure S2 **Transfection of mir-208a into myocardium was monitored by a dissecting fluorescence microscope as shown in green color.**
(TIF)
