# Supplementary figures and images for: MicroRNA-208a Increases Myocardial Fibrosis via Endoglin in Volume Overloading Heart
Source: PLoS One. 2014 Jan 2;9(1):e84188. doi: 10.1371/journal.pone.0084188 (PMC3879305; doi:10.1371/journal.pone.0084188)

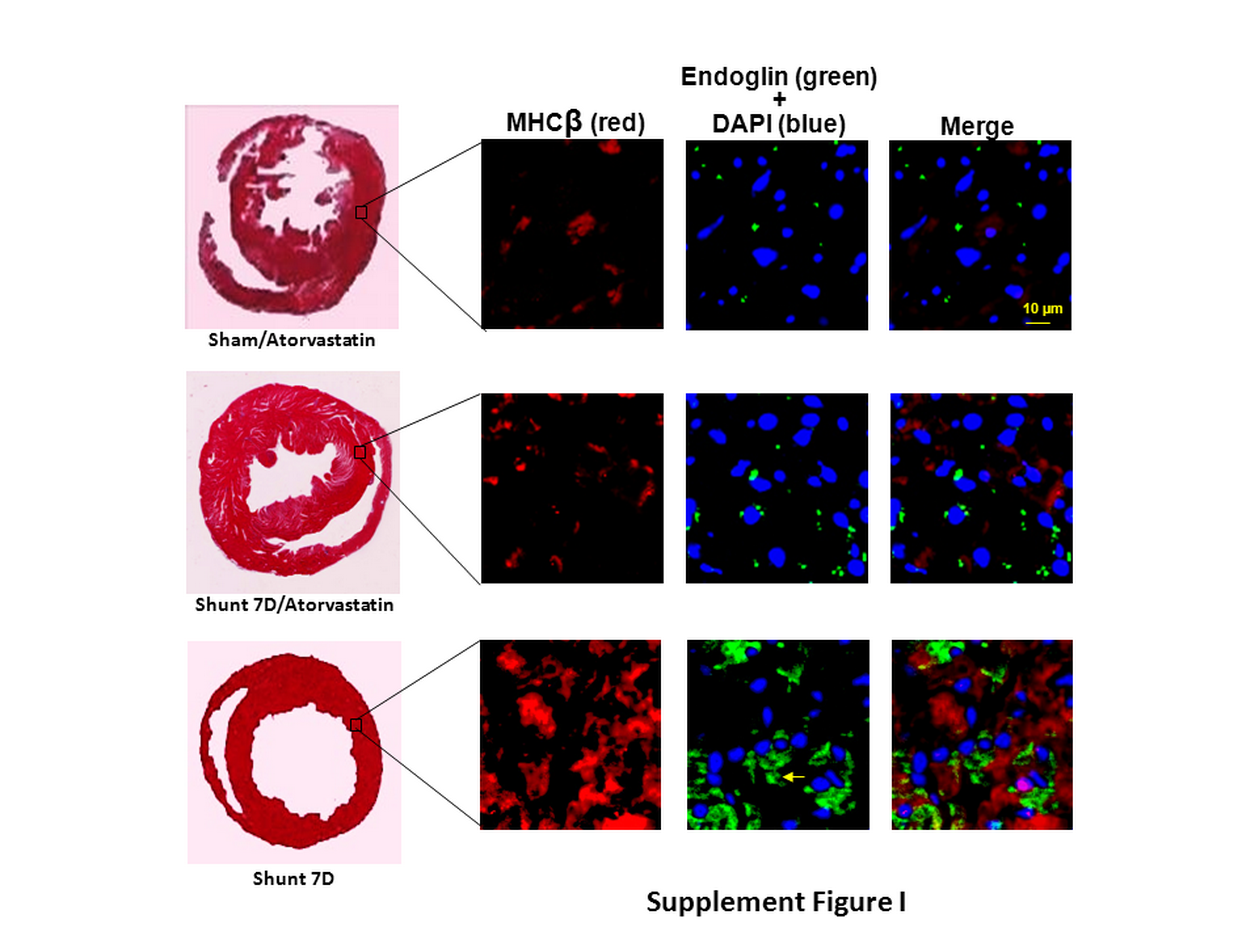

Supplement: Figure S1 — Immunohistochemnical staining of left ventricular myocardium after induction of aorta-caval shunt with or without atorvastatin treatment. There are significantly increased immunoreactive signals for endoglin and MHCβ after AV shunt for 7 days. Pretreatment with atorvastatin significantly decreased the immunoreactive signal induced by AV shunt. Rare endoglin signals were seen in the sham group. (TIF) [file pone.0084188.s001.tif]

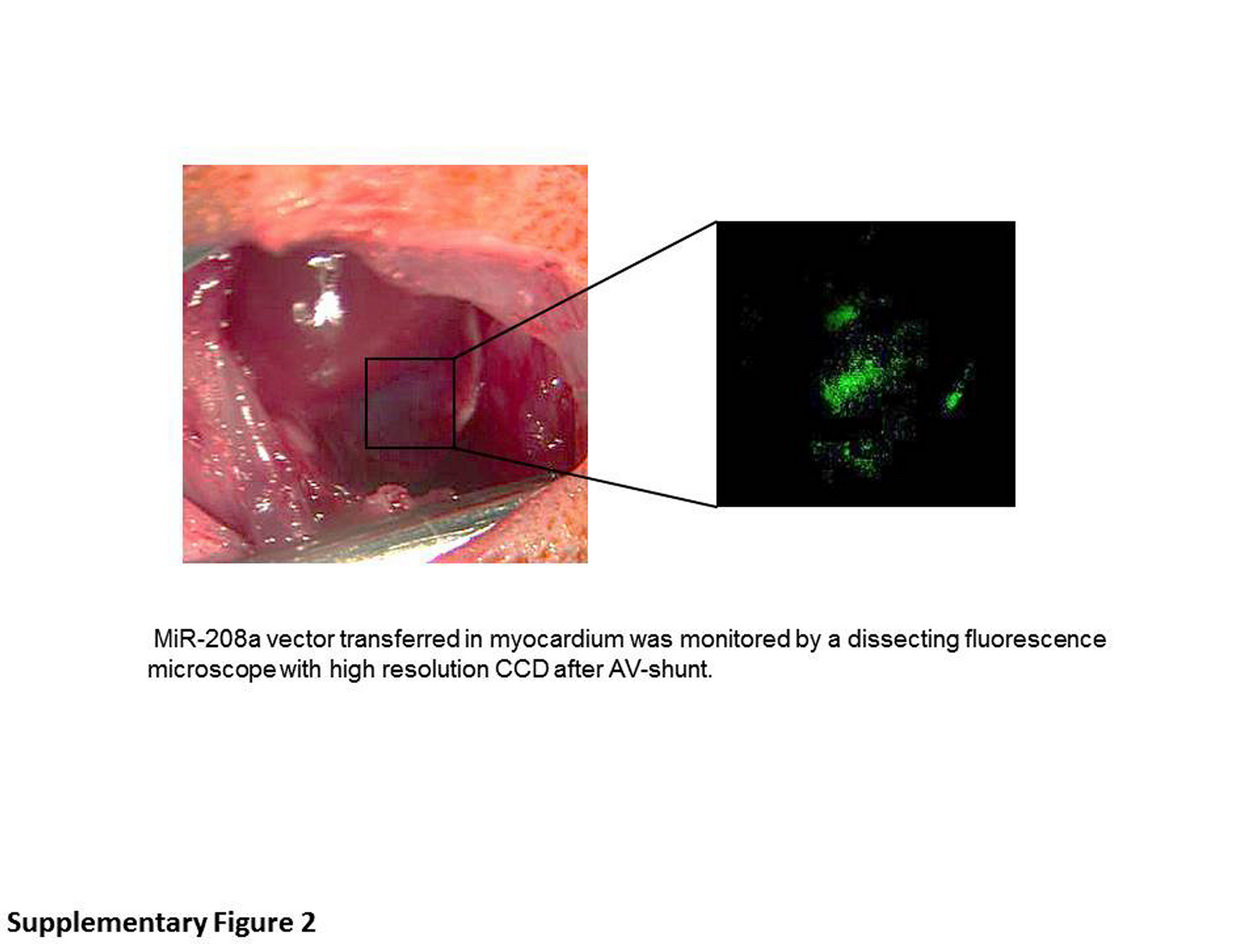

Supplement: Figure S2 — Transfection of mir-208a into myocardium was monitored by a dissecting fluorescence microscope as shown in green color. (TIF) [file pone.0084188.s002.tif]
